# A Bibliometric Perspective on AI Research for Job-Résumé Matching

**DOI:** 10.1155/2022/8002363

**Published:** 2022-10-03

**Authors:** Sergio Rojas-Galeano, Jorge Posada, Esteban Ordoñez

**Affiliations:** ^1^Universidad Distrital Francisco José de Caldas, Bogotá, Colombia; ^2^Natura Software, Bogotá, Colombia

## Abstract

The search for the right person for the right job, or in other words the selection of the candidate who best reflects the skills demanded by employers to perform a specific set of duties in a job appointment, is a key premise of the personnel selection pipeline of recruitment departments. This task is usually performed by human experts who examine the résumé or *curriculum vitae* of candidates in search of the right skills necessary to fit the vacant position. Recent advances in AI, specifically in the fields of text analytics and natural language processing, have sparked the interest of research on the application of these technologies to help recruiters accomplish this task or part of it automatically, applying algorithms for information extraction, parsing, representation, and matching of résumés and job descriptions, or sections within. In this study, we aim to better understand how the research landscape in this field has evolved. To do this, we follow a multifaceted bibliometric approach aimed at identifying trends, dynamics, structures, and visual mapping of the most relevant topics, highly cited or influential papers, authors, and universities working on these topics, based on a publication record retrieved from Scopus and Google Scholar bibliographic databases. We conclude that, unlike a traditional literature review, the bibliometric-guided approach allowed us to discover a more comprehensive picture of the evolution of research in this subject and to clearly identify paradigm shifts from the earliest stages to the most recent efforts proposed to address this problem.

## 1. Introduction

Hiring the right person for a job has become a challenging and time-consuming task for companies today, due to the increasing growth of online recruiting platforms and candidates. Since the acquisition of talent is a crucial aspect of business success by employers, the selection of résumés submitted by a large number of applicants, the extraction of relevant information, and the proper match against the skills required for the job are not a trivial task to perform manually. Therefore, the modern recruiting industry needs to automate the application filtering and classification process and that is where recent advances in text mining (applied to the unstructured text contained in a given *résumé*) can help.

Text mining (or text analytics) is a set of computational tools to transform and analyse unstructured (or freestyle) text found in documents, using algorithms for natural language processing (NLP) and machine learning (ML). By processing large amounts of this kind of data, these algorithms are able to perform tasks such as text categorisation, classification or summarisation, sentiment analysis, entity extraction, and concept identification [1]. For these reasons, text mining is becoming a core technology to develop useful tools in the context of job-résumé matching applications.

In this sense, the aim of this work was to better understand how research on the subject of computer-assisted job-résumé matching has progressed, in particular with respect to the application of text mining methods. To do this, we will analyse the scientific literature published in the last two decades on this topic, following a bibliometric approach aimed at discovering the dynamics of relevant trends, the growth of research production, publication timelines, citation of papers and authors, thematic development, and emerging structures of conceptual, social, and authorship patterns, which together shape the evolution of its research landscape. In this way, we hope to capture a broad overview from initial to more advanced approaches, as well as emerging avenues for current and future work.

The study is organised as follows. An overview of the analytics workflow followed to conduct the study is presented in Section 2, along with the description of materials and methods. The results of each stage in the workflow with a narrative review of selected works identified as a result of such critical analysis are reported in Section 4. The last section presents some conclusions and ideas for future work.

### 1.1. Contributions

This study analyses the literature that addresses the application of text mining to job recruitment, with a focus on the task of job-résumé matching. Unlike a traditional literature review, the bibliometric approach allows us to offer a multifaceted assessment of the evolution of research frontiers on this subject. In particular, we describe the following aspects:

- Performance indicators of publication dynamics from a historical perspective, including trends of frequent terms, productivity, and citation scores. In this facet, the general behaviour seems to indicate that this is an emerging field of research that is actively growing and coinciding with the recent boom in AI technologies for NLP.
- Authorship patterns, leading contributors, institute distribution, and geographic covering of the published research. Here, we identify the seminal papers that introduced the job-résumé matching problem back in 2006 and 2012, and also how there is evidence of a renewed spark of interest since 2016, with institutions from China, India, and the Middle East leading the publication record since then, an indication of the greater relevance of this topic given the large labour markets that characterise these economies.
- Topic maps and thematic evolution of the main concepts covered in the literature, including identification of periods of different research development. In this respect, we found how closely related this problem is to the areas of NLP and ML, concretely to applications such as recommender systems. In addition, we show how approaches to the topic have shifted as AI technology and terminology have evolved, migrating from information extraction, ontologies, and data mining towards, more recently, NLP, neural networks, contextual embeddings, and deep learning.
- Collaboration and intellectual networks emerging from the literature. The results of our social network analysis corroborate the relevance of previously identified highly cited works and authors and also reveal close collaborations not only between academic institutions but also with AI-based industries.
- Selection and review of pertinent works highlighting the most significant contributions to the subject of job-résumé matching during our observation period. This narrative literature review arises as a follow-up to further extend the findings identified with the aforementioned bibliometric analysis.

## 2. Materials and Methods

### 2.1. Data Collection

To conduct our analyses, we initially collected related publication metadata from the Scopus bibliographic database, which is one of the largest citation and abstract information services. Our search was based on connecting three concepts relevant to our objective: the task of interest (*matching*) and the two inputs needed to perform the task (*job description* and *résumé*). Since we were not aware of the exact origin of this research subject, the observation window was left open. Therefore, we extracted bibliographic records using the following search equation, which retrieved a total of 121 documents (as of June 21, 2021)

  TITLE ((“matching” OR “pairing” OR “comparing” OR “searching” OR “parsing” OR “classification” OR “extraction” OR “recommendation” OR “recognition”) AND (“cv” OR “curriculum vitae” OR “resume” OR “résumés” OR “rsum” OR “rsums” OR “skills” OR “employment”) AND (“job” OR “job opening” OR “job post” OR “job description” OR “personnel selection” OR “person-job fit” OR “recruitment”)) OR KEY ((“matching” OR “pairing” OR “comparing” OR “searching” OR “parsing” OR “classification” OR “extraction” OR “recommendation” OR “recognition”) AND (“cv” OR “curriculum vitae” OR “resume” OR “résumés” OR “rsum” OR “rsums”) AND (“job” OR “job opening” OR “job post” OR “job description” OR “personnel selection” OR “person-job fit” OR “recruitment” OR “employment”))

These results were carefully examined by a domain expert who ruled out duplicate and irrelevant records, resulting in a short list of 64 relevant bibliographic entries. The inclusion criteria verified that the study included any of the following AI-related terms in its abstracts: NLP or natural language processing, machine learning or ML, data mining, text mining, deep learning, classification, grouping, regression, ontology, big data, information extraction, information retrieval, neural network, recommendation or recommender system, analytics, or any particular algorithm in the AI domain that was known to the expert.

Furthermore, to validate the reliability of the dataset initially collected, as well as to extend our search to the scientific literature not included in Scopus (nonindexed journals, conference proceedings, theses, and preprints), we used Google Scholar as a complementary bibliographic material source. The following search equation used in Google Scholar returned about 19,300 results (again, as of June 21, 2021)

  Resume job post CV matching

To make the manual examination feasible, the search was broken down into date intervals 2021, 2019-2020, 2017-2018, 2015-2016, and before 2015; at each interval, the expert examined the first 50 results and corroborated or added them to the initial list. In this way, 49 bibliographic records were added to the curated bibliographic collection, which increased to a total of 113 records.

The raw data were then exported to a BIB file [2], a common format used by reference management software such as Zotero or Mendeley. Each bibliographic record includes the following metadata fields: title, author, abstract, keywords, journal, volume, pages, year, publisher, language, document type, citations, affiliation, references, and source (Scopus or Scholar). The dataset is available at https://github.com/Sargaleano/job-resume-lit-rev [3]. No additional preprocessing of the raw data was made.

### 2.2. Bibliometric Methods

**Figure 1:**
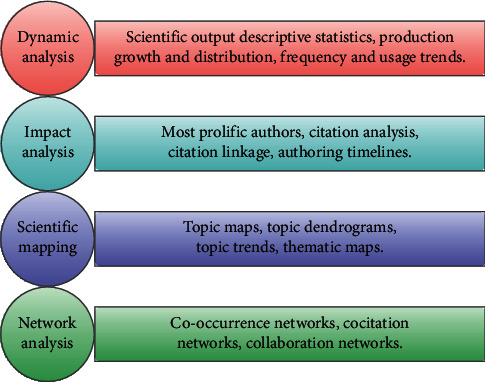
A conceptual framework of the bibliometric tool used in this study.

On the resulting BIB file, we carried out a multifaceted bibliometric analysis. The techniques used to reveal these facets are described in [4], covering performance bibliometrics and scientific mapping tools such as production descriptive statistics, scientific output growth and impact, document distribution, specialised journal dynamics, prolific authors and institutions, author's timelines, citation impact metrics, historiographic lineage of citations, frequent word and word cloud analyses, topic maps and dendrograms, topic trends, and thematic evolution, in addition to network analyses of co-occurrence, co-citation, and collaboration patterns. A conceptual framework of the bibliometric methods we applied is shown in Figure 1.

Our study was conducted using the *Bibliometrix* [5] software package, an open-source *R* library that deploys the aforementioned bibliometric tools, along with its *Biblioshiny* visual interface, version 3.1.

## 3. Results

In this section, we present the results of the bibliometric-guided literature review on job-résumé matching research. We organise these results in the facets of research production (performance indicators and research trends) and structures of knowledge (conceptual, social, and intellectual emerging structures). After discussing our main findings, we carried out a narrative review of selected relevant works focusing on the application of NLP approaches to the job-résumé matching problem.

### 3.1. Research Dynamics

We start with some of the research dynamic performance indicators for the collected dataset, as summarised in Table 1. A total of 113 documents were analysed, with an average number of 10.65 citations (1.42 per year), which is comparatively low with respect to relevant categories such as artificial intelligence and informetrics, as reported in [6]. On the other hand, the number of keywords used to categorise the papers, either by the authors or assigned from a thesaurus, is quite high (217 and 571, resp.), which suggests that this area of interest is not yet clearly delimited. The average age of the articles is 3.75 years, which seems to indicate that most of the scientific production in this field, along with a spark of interest from the research community, is relatively recent.

**Figure 2:**
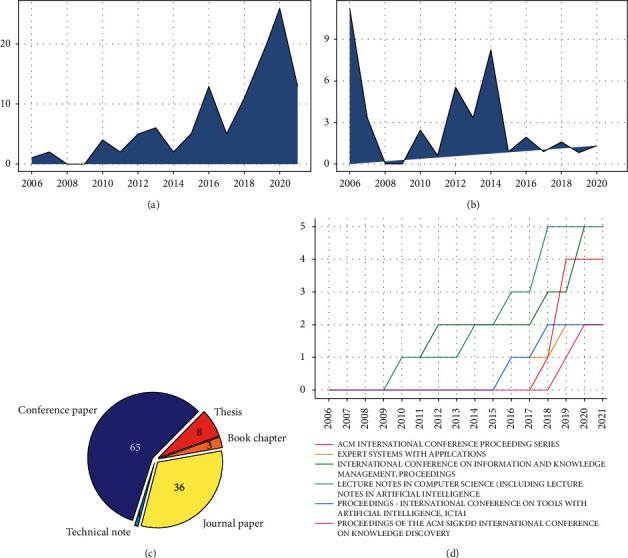
Research production dynamics. (a) Annual scientific production. (b) Average citations per year. (c) Document type distribution. (d) Annual source growth.

Now, in terms of structure statistics, 342 unique authors were found (372 author appearances in total), which indicates that the academic community interested in the subject is proportionately broad, and this is corroborated by the high number of unique authors per document (3.03 or 342/113). Similarly, the average coauthorship and the collaboration index [7], which are 3.29 and 3.34, respectively, conform to the average in related categories such as artificial intelligence or informetrics, as reported in [6]. In addition, Figure 2 shows an alternative view of the performance indicators based on trends. The graph of annual scientific production (Figure 2(a)), which corresponds to an average growth rate over time of 14.45%, exhibits a constant increase since 2017, reaching a peak in 2020 of 24 papers published. Furthermore, the number of citations in Figure 2(b) is higher in papers published between 2012 and 2014, perhaps because the most recent works have not yet matured enough so as to accrue larger citation counts. We observe that these plots show a decrease between 2008 and 2009 since no published works were found in that interval.

Regarding the type of production, we highlight that the preferred means of dissemination are the proceedings of academic conferences (56% or 65/113), followed by scientific journals (32% or 36/113) and postgraduate theses (7% or 8/113).

The former suggests the predominance of exploratory research that seeks rapid communication of results, for which conferences seem to serve better. In this sense, there is a growing preferential trend towards publications in conferences related to computer science, data mining, and artificial intelligence, which seems to become the natural academic setting for this subject.

**Figure 3:**
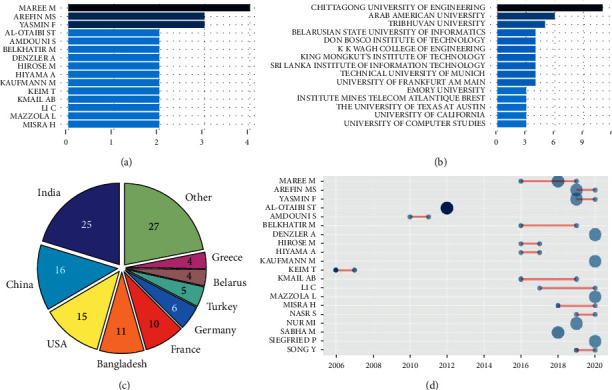
Authoring dynamics. (a) Most prolific authors. (b) Most prolific institutions. (c) Country of origin distribution. (d) Authors' timelines.

Besides, the analysis of the authorship dynamics is shown in Figure 3. Two interesting facts can be pointed out: first, the problem has been addressed mainly by authors and university faculties from Asian countries (India, China, Bangladesh) followed by the United States and some European countries. Second, the authoring timelines show that before 2016 activity was low although some very influential papers were published (large dark blue dots), whereas most of the production of prolific authors started from 2018.

**Figure 4:**
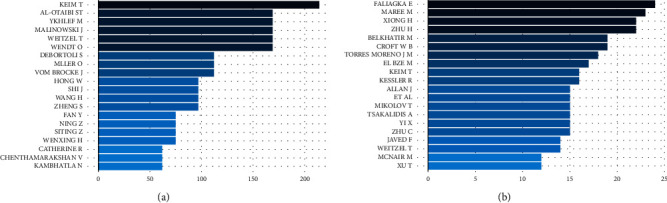
Most cited authors. (a) Overall. (b) Collection.

In addition, we also conducted an impact analysis based on the most cited papers and authors. These are shown in Table 2 and Figure 4, as a result of external citations reported by bibliographic databases or calculated from references within the collection.

We emphasise that many of the top external authors and papers cited, incidentally, are also highly cited within the collection; we reason that those papers can be chosen as the initial basis for a review of the state-of-the-art literature on job-résumé matching.

**Figure 5:**
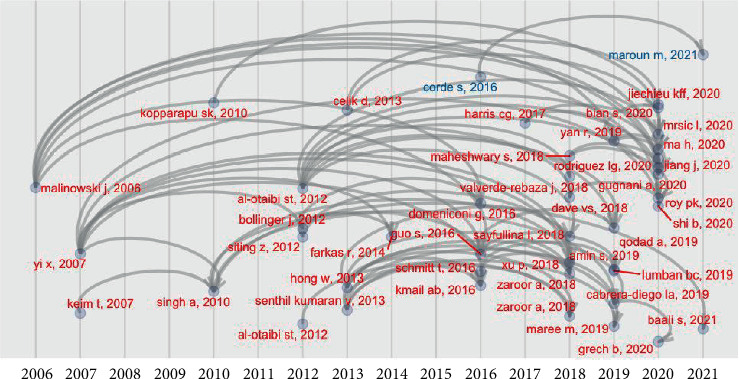
Historiographic lineage of citations.

We completed the analysis of the research dynamics with the historiograph, i.e., the diagram of historical direct citation linkages between papers within the collection (see Figure 5). Here, the pattern of citation dynamics begins in 2006 with seminal articles from [8, 9]; then, after a lapse of 5–6 years, other influential papers appeared such as [10–12, 19, 29]. Then, there is a steep slope of increased production in recent years as a thriving body of publications citing these articles becomes apparent. We believe that it is also worth including those seminal articles in the relevant set of articles in the literature review.

### 3.2. Conceptual Structures

**Figure 6:**
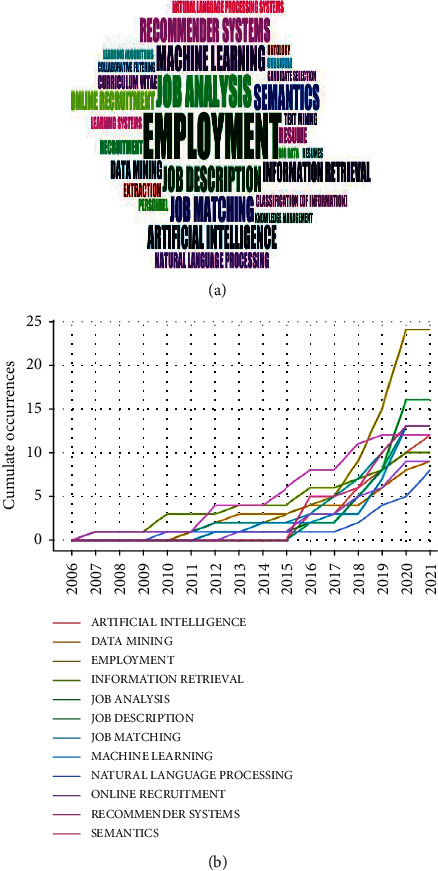
Analysis of keyword usage. (a) Keywords cloud. (b) Most frequent keywords.

**Figure 7:**
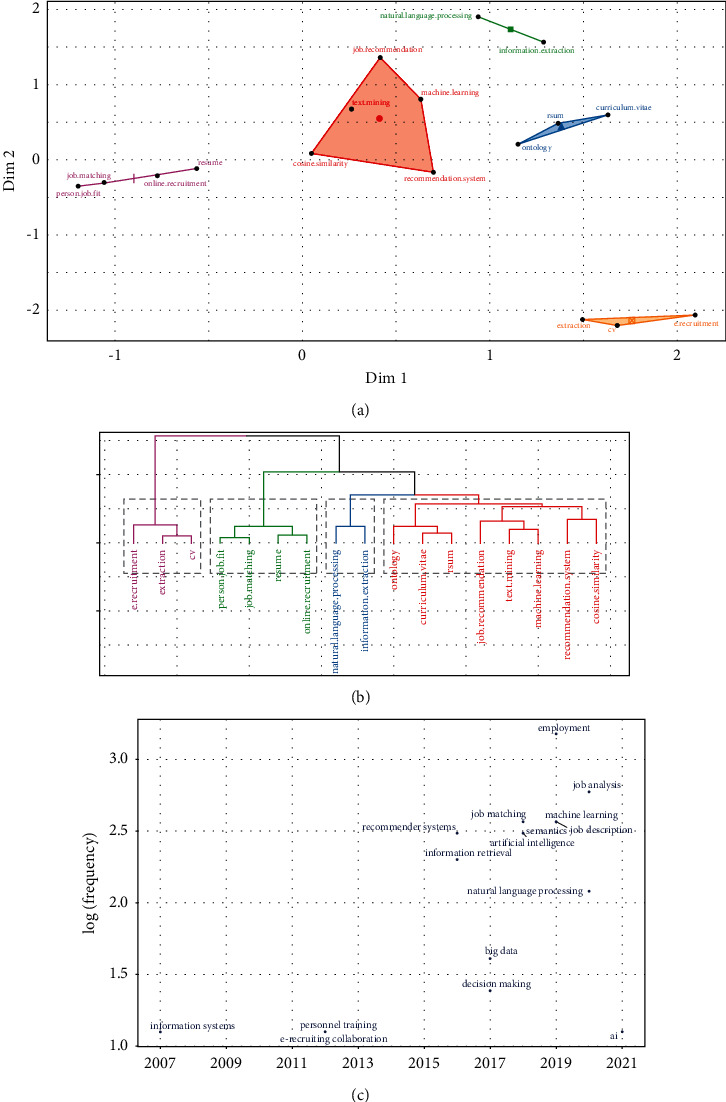
Analysis of topic evolution. (a) Topic map. (b) Topic dendrogram. (c) Topic trends.

In this facet, we initially present the results of the analysis of the use of keywords (Figure 6) and the evolution of the research topics (Figure 7). In addition, a word cloud of the most prominent keywords (Figure 6(a)) exhibits two categories that stand out, one related to the subject matter (*employment, job analysis, job description, job matching, online recruitment and résumé*, among others) and the other with a wide variety of AI topics that have been proposed to address the problem (*machine learning, recommender systems, artificial intelligence, data mining, information retrieval, natural language processing, etc.*).

The temporal trends of appearance of these keywords are shown in Figure 6(b); here, the preponderance of terms such as *machine learning* and *artificial intelligence* can be seen rising since 2018, suggesting these as the appropriate fields of knowledge to tackle this problem.

Next, we carry out an analysis of the research topics on the job-résumé matching problem (Figure 7). The map in Figure 7(a) shows a set of conceptual topics that encompass relevant keywords whose proximity, calculated through dimensionality reduction and clustering techniques on the document-term frequency matrix, is projected in a 2D plane comprising the two coordinates with the greatest variability of shared usage between documents [5].

As a result of this analysis, five topics of scientific relevance are distinguished: the topic of automatic talent recruitment (purple color), the topic of artificial intelligence aspects (red color), the topic of natural language processing approaches (green color), another one related to the semantic ontologies underlying the curriculum vitae (blue color), and a topic (orange color) that groups terms such as information extraction and recruitment again, which could eventually be merged with the purple topic.

In fact, these themes can be alternatively visualised as a hierarchical tree or dendrogram (Figure 7(b)), in which proximity is represented by hierarchical relationships between concepts, producing different vertical partitions corresponding to the fusion or segregation of the topics aforementioned. In addition, the figure includes a topic trend analysis (Figure 7(c)), which shows the timely variation of usage trends. It is observed that at the beginning of the study in this area the problem was associated with *information systems*, *personnel training,* and *e-recruitment*. As the research progressed, in mid-2017 and in more recent years, the term *job matching* began to be used to refer to the problem and it also began to be identified within the study area of *natural language processing*, *artificial intelligence,* and *machine learning*.

**Figure 8:**
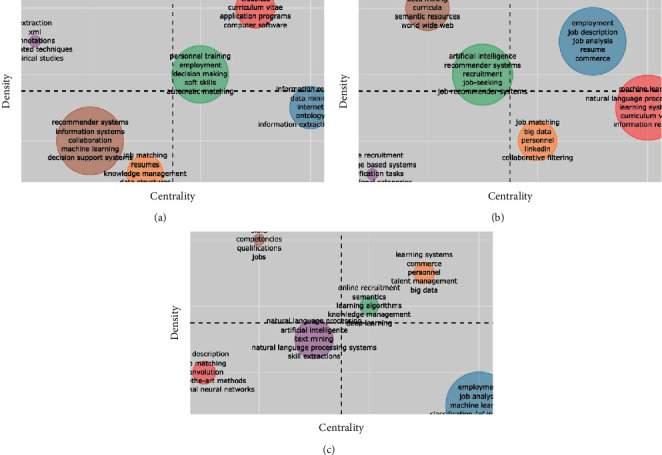
Analysis of thematic evolution. Quadrants counterclockwise represent motor themes (first), highly specialised themes (second), emerging themes (third), and fundamental themes (fourth). (a) Thematic map (2006–2015). (b) Thematic map (2016–2018). (c) Thematic map (2019–2021).

An alternative view of the emergence of topics of interest can be obtained through the evolution of thematic maps as shown in Figure 8. A thematic map [30, 31] is a 2D projection of trend topics, whose dimensions are centrality (relevance of a topic in the research field) and density (maturity in the development of a topic). Thus, the four quadrants of the map (counterclockwise) represent motor themes (first quadrant), highly specialised themes (second), emerging themes (third), and fundamental themes (fourth). Therefore, o visualise the thematic evolution, we divide the observation window into three intervals according to the production growth peaks (Figure 2(a)) mentioned above: 2006–2015 (Figure 8(a)), 2016–2018 (Figure 8(b)), and 2019–2021 (Figure 8(c)).

In this way, we find that during the initial research interval (Figure 8(a)) the motor themes focused on delineating the problem itself (terms such as *curriculum vitae, employment, automatic matching*), using fundamental tools related to *information extraction*, *ontologies,* and *data mining*; at the time, novel terms such as *recommendation systems*, *machine learning*, and *job matching* were just emerging.

Subsequently in the middle interval (Figure 8(b)), the motor themes moved around *job description and resume analysis* using fundamental approaches related to *machine learning*, *natural language processing,* and *collaborative filtering*; simultaneously, the terms *artificial intelligence* and *job recommendation systems* were used to describe specialised topics.

Lastly, the most recent interval of research (Figure 8(c)) indicates that the current dominant terms for this subject matter are related to *online recruitment*, *talent management,* and *learning algorithms*, whereas technologies associated with *text mining, neural networks, deep learning,* and *natural language processing* are emerging. The latter has seen a renovated interest mostly caused by the recent surge of language models applied to NLP; see e.g., [32].

**Figure 9:**
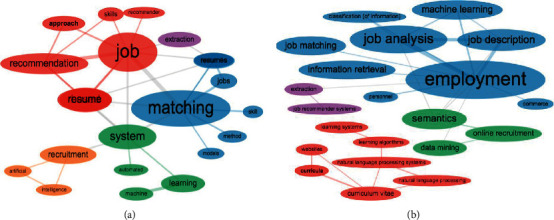
Co-occurrence networks. (a) Title terms. (b) Abstract terms.

To round off the analysis of conceptual structures, we report the term co-occurrence networks of Figure 9, obtained from the titles (Figure 9(a)) and abstracts (Figure 9(b)) of the collection of papers. In contrast to the frequency analysis reported in Figure 6, these two complementary networks depict how those common terms are interrelated, in the sense that they appear close together in titles and abstracts, respectively, when used to describe the problem and proposed solution approaches. Figure 9(a) shows a central region dominated by the terms *job-resume-matching,* which is extended with connections to key aspects of the problem such as *matching skills* or *job recommendation*; furthermore, it also emphasises the reliance on the fields of *artificial intelligence* and *machine learning* through their connection to the term node *systems*.

Likewise, Figure 9(b) highlights the broader term *employment* as the central node of the main cluster of the network, interconnected with terms such as *job analysis* and *job description*; besides, another more specialised cluster can be seen here, relating the term *curriculum vitae* with *natural language processing*, perhaps confirming an emerging structure of the most recent approaches to address the problem, as mentioned before (Figure 8(c)).

### 3.3. Social Structures

**Figure 10:**
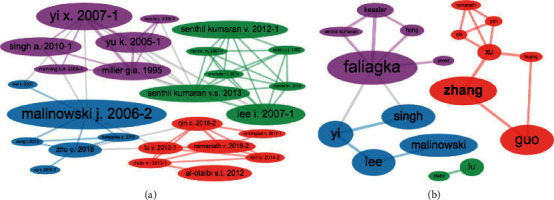
Co-citation networks. (a) Papers. (b) Authors.

We begin this section by examining the co-citations networks reported in Figure 10. The network of paper co-citations (Figure 10(a)) reveals four clusters whose central nodes are either seminal works [8, 9, 12] or highly cited review papers [10], which is consistent with our previous observations (see Table 2 and Figure 5). The clusters suggest these can be interesting works to be considered in an extended literature review.

Furthermore, Figure 10(b) network shows clusters of frequently co-cited authors, presumably sharing research interests related to the domain problem. Together with the results of authoring dynamics (Figure 3), this network is useful to identify body of works of some candidate authors also worth considering in a prospective extended review.

**Figure 11:**
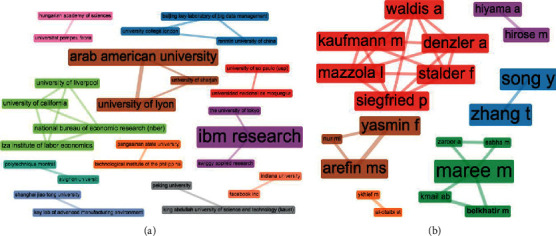
Collaboration networks. (a) Institutions. (b) Authors.

Likewise, extra clues suggesting other authors or institutions working on the job-résumé problem, which may be worth reviewing, can be found on the collaboration networks revealed in Figure 11. We note these collaboration structures are intended to provide complementary information for the analysis of authorship dynamics of Figure 3.

### 3.4. Related Work

While examining the collection of documents retrieved for our study, we found related works reporting literature reviews on the job matching problem (these were identified because of the use of the terms *review* or *survey* within the title or keywords). Therefore, we carry out a closer look at these works, which we describe below in chronological order.

A survey on job recommendation systems was conducted in [19]; initially, they characterise the problem of job-person matching by defining aspects of the user profile, the extraction of characteristics from the curriculum, and the similarity measures for job descriptions and user profiles. They also list approaches to solve this problem using recommender systems (content-based or collaborative). Lastly, they describe some technologies implemented to provide online recruiting services (e.g., CASPER, Bilateral Recommender, Proactive, Absolventen). This study can be a useful entry point for understanding the basic concepts and also to provide practitioner-oriented advice rather than a rigorous description of research progress on this subject. Furthermore, it turns out to be an influential article (with 75 citations at the time of writing), as it is shown in Figure 4(a), Figure 5, and Table 2(a); however, no further works by these authors on this topic were found afterwards. Nonetheless, [33] recently reported a related paper by the same title, which updates some of the aspects covered by [19], while expanding the discussion to major issues in job recommendation systems (shortages, scalability, overspecialisation, spam attacks, etc.), as well as extending the review to papers addressing this problem published after 2012.

Similarly, the work of [10] also reports a survey of job recommendation systems, with a focus on e-recruitment platforms. They describe a detailed characterisation of hybrid, collaborative, knowledge-based, and content-based recommendation systems. The context of candidate recommendation for a job is presented, comprising the extraction of characteristics (from the candidate's profile and the job description) and the ranking of candidates by similarity measures. In contrast to the aforementioned survey, this work provides a more exhaustive taxonomy of the proposed approaches with relevant references to the literature up to 2011, which better illustrates the research front on the job-person fitting problem at that time. Incidentally, this is also an influential paper with 144 citations (when we retrieved the collection, see Table 2(a) and Figure 4(a)) and it has been subsequently referenced in multiple works (Figure 5) although not by the same authors.

On the other hand, the paper by [34] focuses on *parsing* the different sections of the semi-structured CV document (keyword, grammatical and statistical approaches). In addition to describing a method for résumé screening and ranking, this study includes a brief review of the literature centred on query parsing and searching techniques, as well as job-résumé matching mainly based on ontologies, such as the work of [35]. A recent work by [36] takes a practitioner perspective on the broader topic of AI-assisted HR recruitment. It highlights how AI technology is driving processes in the different stages of talent acquisition, improving speed and efficiency in the search for the most suitable candidates for the jobs. To do this, they based the review on management and business magazines and websites, highlighting applications such as chatbots to automate candidate interviews, which are currently being used by large multinational companies.

Also recently, [37] reported on a review of 105 articles to identify the progress of machine learning applications in HR analytics. We highlight that some interesting network analyses were carried out to relate ML and data mining concepts and algorithms with HR functions. In this sense, this study is closer to our work, since we also perform network analysis to visualise associations between various aspects of research in the job-résumé matching problem. However, our study focuses its scope on this task, while [37] addressed a wider picture of HR functions (recruitment, selection, assignment, training, participation, etc.) and also differs in the depth of analysis as we perform a broader set of bibliometric techniques, which include various types of network analysis (co-occurrences of keywords, co-citations of papers and authors, collaboration networks and topic maps, among others).

### 3.5. Narrative Review of Selected Works

The ultimate purpose of the described bibliometric analyses is to help identify the most relevant topics, papers, authors, and trends in the research landscape of the subject matter, to properly target a literature review. Therefore, based on our findings we selected the works of interest listed in Table 3, to perform a narrative review of research on the job-résumé matching problem, as presented below.

We start with the work of [9], which identifies the job-resumé matching problem as a suitable task for recommendation systems, where candidate skills and recruiters' job demands are matched so as to make CV recommendations, much like the way products are recommended to buyers on e-commerce platforms. The recommender method uses two probabilistic latent models, with visible variables that include job descriptions and skills of the candidate, and output variables indicating the suitability of a candidate or job; the model parameters are estimated using the expectation-maximisation algorithm.

A follow-up paper by [23] also describes the same probabilistic model approach for the CV recommender and the job recommender, extending its interpretation to a broader and multilayered framework of suitable collaboration partners, where not only individual or unary attributes are taken into account but also relational or binary attributes between seekers and providers are taken into account.

In the work of [8], a different point of view is proposed, addressing the problem of job-person matching using structured relevance models. These models perform queries over the flattened structures of résumés and job descriptions using a set of relevant labeled résumés to retrieve similar ones from the larger collection of unlabelled résumés.

On a different note, [13] introduced a support tool to help shortlist the right résumés for job openings. The system first processes the unstructured information contained in the curriculum by means of a table analyser, a segmenter, and a concept recogniser; these are used to extract a set of features that include text-related, visual, and lexical attributes. In addition, a conditional random field model performs named entity recognition. In this way, a total of 37 features are extracted. Then, the tool classifies the candidates through a scoring model that applies TF-IDF (term frequency, inverse frequency of documents) on a query created from the job requirements and the features extracted from the collection of résumés. This is one of the first intelligent systems intended to help recruitment professionals filter job applications.

Subsequent works in Table 3 are the surveys conducted by [10, 19] that we reviewed in the previous section. Next on the list is the paper by [12], which proposes an intelligent tool to rank job applicants using ontology mapping. In their system, both candidate résumés and job descriptions are represented as ontologies that are cross-matched to retrieve the most suitable candidates. Matching scores are calculated with a set of rules to obtain similarities between corresponding categorical or numeric features within the ontologies.

Taking a different approach, [38] proposes a recruitment tool to rank job applicants. For this purpose, they developed a ranking module that extracts relevant information from the candidate's LinkedIn profile (e.g., whether or not a certain skill is found) and uses machine learning algorithms (linear regression, regression tree, and support vector regression) to provide a suitability score. A complementary module computes a personality trait score, applying a linguistic analysis of the blogs written by the candidates using a linear regression model trained with historical data. The authors remark that the tool is intended to assist, not replace, expert recruiters in their decision-making process.

Next, in the work of [11], a comparative study of online job recommender systems (CASPER, Proactive, PROSPECT, eRecruiter) is conducted, contrasting their different approaches (content, collaborative filter, knowledge, reciprocal recommendations, or bilateral and hybrid approaches). Then, they address the large-scale challenge facing China's public employment office in matching candidates with job offers. To do this, they developed an online platform and propose a clustering approach in which users are grouped according to some attributes related to user information, activity, and behaviour within the platform. In this way, active, passive, and moderate user clusters are found, and based on these categories, the jobs are filtered using the most appropriate strategy of the recommendation techniques mentioned above.

On the other hand, the work of [15] describes a job-résumé matching system, which analyses unstructured résumés and job descriptions to extract and assign labels to tokens using regular expressions and pattern matching techniques. The resulting job and résumé models are then compared using domain-specific ontologies and ontology similarity measures.

Likewise, [40] proposes a system that integrates a knowledge base of occupational categories to classify job openings and résumés and to perform their respective matching. The system analyses the unstructured documents using a series of NLP steps, including section splitting, n-gram tokenisation, stop-word removal, part-of-speech tagging (POST), and named entity recognition (NER). Skills extracted in this way from both résumés and jobs are classified into occupational categories according to the skill knowledge base. Finally, the match score is based on semantic similarities computed from ontologies and statistical measures of concept relationship. An extended detailed description of this approach is provided in [39], emphasising the formalities of the semantic network construction procedure for the job and résumé models, along with additional similarity metrics used for the matching.

Another study by [41] also proposes a framework for the ranking of candidates that initially extracts relevant information from résumés via tokenisation and NER using the *spaCy* library. Applicant scores are obtained according to a set of predefined rules about the extracted attributes along with their values and the employer's requirements. The final ranking is done with a skyline filtering method, which selects the nondominated candidates in the Pareto front of the priority criteria defined by the employer. The authors evaluated different divide and conquer and block nested loop algorithms for this purpose, while SQL query and map reduction techniques were considered within the same framework in the related work by [42].

A more recent study by [43] considers the similar problem of recommending educational programs based on the CV and personal preferences of candidates. To that end, they use explicit semantic analysis (ESA), a technique that represents domain-specific semantic concepts based on the Wikipedia entry pages related to that domain. A knowledge base of such concepts is represented as a numeric matrix in which each row is associated with a concept and each column represents the root words found within the collection of pages. Each row is computed as the corresponding bag of words or TF-IDF representation of the concept. Hence, for an arbitrary query document (a candidate's résumé) a ranking of related concepts is obtained by comparing the similarity between its root vector representation and the rest of root vectors of concepts in the knowledge base. This is the first study that applies the distributed representation of documents to perform the person-job matching within the documents reviewed so far.

In contrast, the work of [44] describes a model for a job matching application to be used in a government employment agency, which also applies vectorization. The similarity between vacancies and candidates is based on the cosine distance of their respective vector representations. However, in this approach, the vectors consist of values of predefined attributes (title, educational qualification, experience, age, gender, etc.) that are retrieved from the collection of résumés transformed into the standard layout used by the agency.

Finally, the study by [45] proposes a classification method to help candidates identify their most suitable level of qualification for job vacancies and also to help recruiters filter and rank the most promising applicants for a job. Its underlying model uses novel transformer-based language encoders to provide distributed vector representations of both résumés and job descriptions, which take into account contextual features in their content. To test their models, a careful manual annotation process of a large batch of résumés for clinical positions was carried out, including inter-annotation agreement of opinions by three different experts. They then use the dataset to experiment with two ML tasks: multi-class classification at the résumé proficiency level and job-résumé matching as a binary classification task.

An interesting aspect discussed in this last study is that since the transformer model accepts short texts as inputs with a limited number of tokens (words), the authors evaluate various strategies to encode the full length of the different sections that make up a résumé document, including section trimming, section pruning, chunk segmenting, section encoding, and multi-head attention layers. All of these strategies use some type of vector aggregation of the contextual embeddings produced by the transformer model for each unit of analysis. This is the first paper in our review that applies pretrained contextual language models to the job-résumé matching problem. As such, it also provides pointers for recent works in the literature considering the use of deep, convolutional, and transformer neural models, as well as other word embeddings for résumé classification.

## 4. Concluding Remarks

In this study, we show how the insights gained from the multifaceted bibliometric analysis are useful to discover a broader panorama of the research front in the problem of job-résumé matching. We advocate that such a multidimensional perspective yields a more critical and comprehensive guide to discovering the dominant and emerging trends on the subject. If carried out regularly, the workflow can be used to track the progress of any research field over time, providing an interesting tool to assess the dynamics and structures that emerge, evolve, and mature as its scientific literature develops.

In particular, our findings suggest that research on the problem of job-résumé matching has undergone several AI paradigm shifts, starting from information retrieval methods in the early stages (mid-2000 s) that were based on classical NLP techniques for cleaning, stemming, tokenisation, and string regular expression pattern matching, transitioning then to ontology semantic analysis (early 2010 s), followed by the use of word embeddings (second half of 2010 s), which eventually led to the application of distributed contextual representations in deep neural and transformer models in recent years. We believe that the latter seems to emerge as the most promising avenue of research in the nearest future for this research problem.

## Figures and Tables

**Table 1:** Bibliometric statistics for the collected dataset.

Dynamic indicators		Structure indicators	
Timespan	2006–2021	Authors	342
Documents	113	Author appearances	372
Avg. citations per document	10.65	Single-authored documents	15
Avg. citations per year per doc	1.42	Authors per document	3.03
Author's keywords	217	Coauthors per document	3.29
Keywords plus	571	Collaboration index	3.34
Average years from publication	3.75	References	2508

**Table 2:** Most cited papers.

*(a) Overall*		
*(Authors, year)*	*Citations*	
	*Collection*	*Overall*
(Yi et al. 2007) [8]	9	49
(Malinowski et al. 2006) [9]	9	169
(Al-Otaibi et al. 2012) [10]	8	144
(Hong et al. 2013) [11]	5	97
(Senthil and Sankar 2013) [12]	5	46
(Singh et al. 2010) [13]	4	62
(Yan et al. 2019) [14]	3	9
(Guo et al. 2016) [15]	3	26
(Maheshwary and Misra 2018) [16]	2	14
(Celik et al. 2013) [17]	2	9
(Harris 2017) [18]	2	8
(Siting et al. 2012) [19]	2	75
(Schmitt et al. 2016) [20]	2	17
(Cabrera-Diego et al. 2019)[21]	1	2

*(b) Within collection*		
*(Authors, year)*	*Citations*	*Average year*

(Malinowski et al. 2006) [9]	169	10.6
(Al-Otaibi et al. 2012) [10]	144	14.4
(Debortoli et al. 2014) [22]	112	14.0
(Hong et al. 2013) [11]	97	10.8
(Siting et al. 2012) [19]	75	7.5
(Singh et al. 2010) [13]	62	5.2
(Yi et al. 2007) [8]	49	3.3
(Senthil and Sankar 2013) [12]	46	5.1
(Keim 2007) [23]	45	3.0
(Kucel et al. 2016) [24]	31	5.2
(Guo et al. 2016) [15]	26	4.3
(Kopparapu 2010) [25]	25	2.1
(Al-Otaibi et al. 2012)[26]	25	2.5
(Deepak et al. 2020) [27]	24	12.0
(Almalis et al. 2015) [28]	21	3.0

**Table 3:** Selected works for the narrative review.

Title	Reference	Selection criteria
Matching people and jobs: a bilateral recommendation approach	[9]	Highly cited (Table 2), seminal paper (Figure 5), cluster centrality (Figure 10(a), Figure 10(b))
Extending the applicability of recommender systems: a multilayer framework for matching human resources	[23]	Highly cited (Figure 4), seminal paper (Figure 5)
Matching résumés and jobs based on relevance models	[8]	Highly cited (Table 2), seminal paper (Figure 5), cluster centrality (Figure 10(a), Figure 10(b))
PROSPECT : a system for screening candidates for recruitment	[13]	Highly cited (Table 2), seminal paper
Job recommender systems: a survey	[19]	Review paper, highly cited (Table 2), influential paper (Figure 5)
A survey of job recommender systems	[10]	Review paper, highly cited (Table 2), influential paper (Figure 5)
Towards an automated system for intelligent screening of candidates for recruitment using ontology mapping (EXPERT)	[12]	Highly cited (Table 2), influential paper (Figure 5), cluster centrality (Figure 10(a))
Application of machine learning algorithms to an online recruitment system	[38]	Highly cited in collection (Figure 4(b)), cluster centrality (Figure 10(b))
A job recommender system based on user clustering	[11]	Highly cited (Table 2), influential paper (Figure 5)
RésuMatcher: a personalised résumé-job matching system	[15]	Highly cited (Table 2), influential paper (Figure 5), cluster centrality (Figure 10(a))
JRC : a job post and resume classification system for online recruitment, analysis, and shortcomings of e-recruitment systems	[39, 40]	Recent timeline, authoring dynamics (Figure 3), highly cited in collection (Figure 4(b))
Developing a framework for potential candidate selection, potential candidate selection using information ex-date selection using information extraction and skyline queries	[41, 42]	Recent timeline, authoring dynamics (Figure 3), ML or NLP keywords (Figure 6)
A domain-specific ESA method for semantic text matching	[43]	Recent timeline, authoring dynamics (Figure 3), ML or NLP keywords (Figure 6)
A vectorisation model for job matching application of a government employment service office	[44]	Recent timeline, authoring dynamics (Figure 3), ML or NLP keywords (Figure 6)
Competence-level prediction and resume and job description matching using context-aware transformer models	[45]	Recent timeline, authoring dynamics (Figure 3), ML or NLP keywords (Figure 6)

## Data Availability

The data used in the analysis is actually a collection of bibliographic metadata, which has been made publicly available at: https://github.com/Sargaleano/job-resume-lit-rev [3].
